# Supplementary material for: Decoding the genetic architecture of hernia through genome-wide association and multi-trait analyses
Source: medRxiv. 2026 May 15:2026.05.12.26353033. Preprint. [Version 1] doi: 10.64898/2026.05.12.26353033 (PMC13193034; doi:10.64898/2026.05.12.26353033)
Supplement: Supplement 1 [file NIHPP2026.05.12.26353033v1-supplement-1.pdf]

medRxiv preprint doi: https://doi.org/10.64898/2026.05.12.26353033; this version posted May 15, 2026. The copyright holder for this preprint (which was not certified by peer review) is the author/funder, who has granted medRxiv a license to display the preprint in perpetuity.
It is made available under a CC-BY 4.0 International license .

Supplementary Material

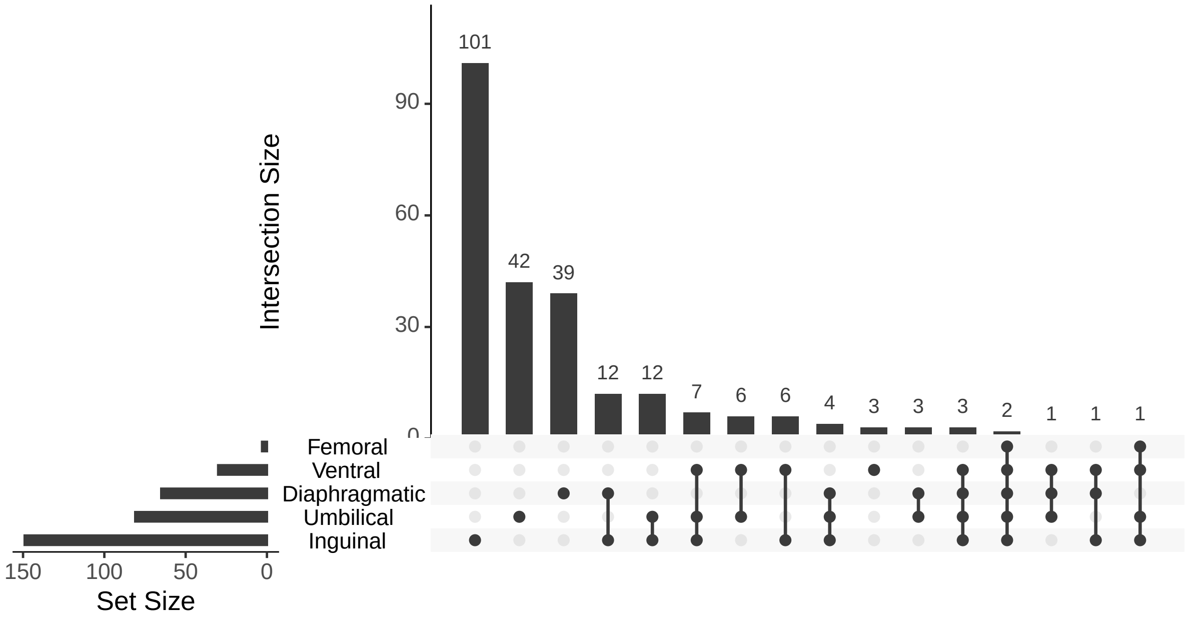

Supplementary Figure 1: Upset plot demonstrating the number of shared genome-wide significant loci between each hernia subtype. Genome-wide significant loci were classified as shared between hernia subtypes if the 1 Mb region surrounding a lead variant overlapped with a 1 Mb region surrounding a different lead variant. Inguinal hernia shared the greatest number of loci with umbilical hernia (n = 12) and diaphragmatic hernia (n = 12). Two loci were shared across all five subtypes of hernia.

medRxiv preprint doi: https://doi.org/10.64898/2026.05.12.26353033; this version posted May 15, 2026. The copyright holder for this preprint (which was not certified by peer review) is the author/funder, who has granted medRxiv a license to display the preprint in perpetuity.
It is made available under a CC-BY 4.0 International license .

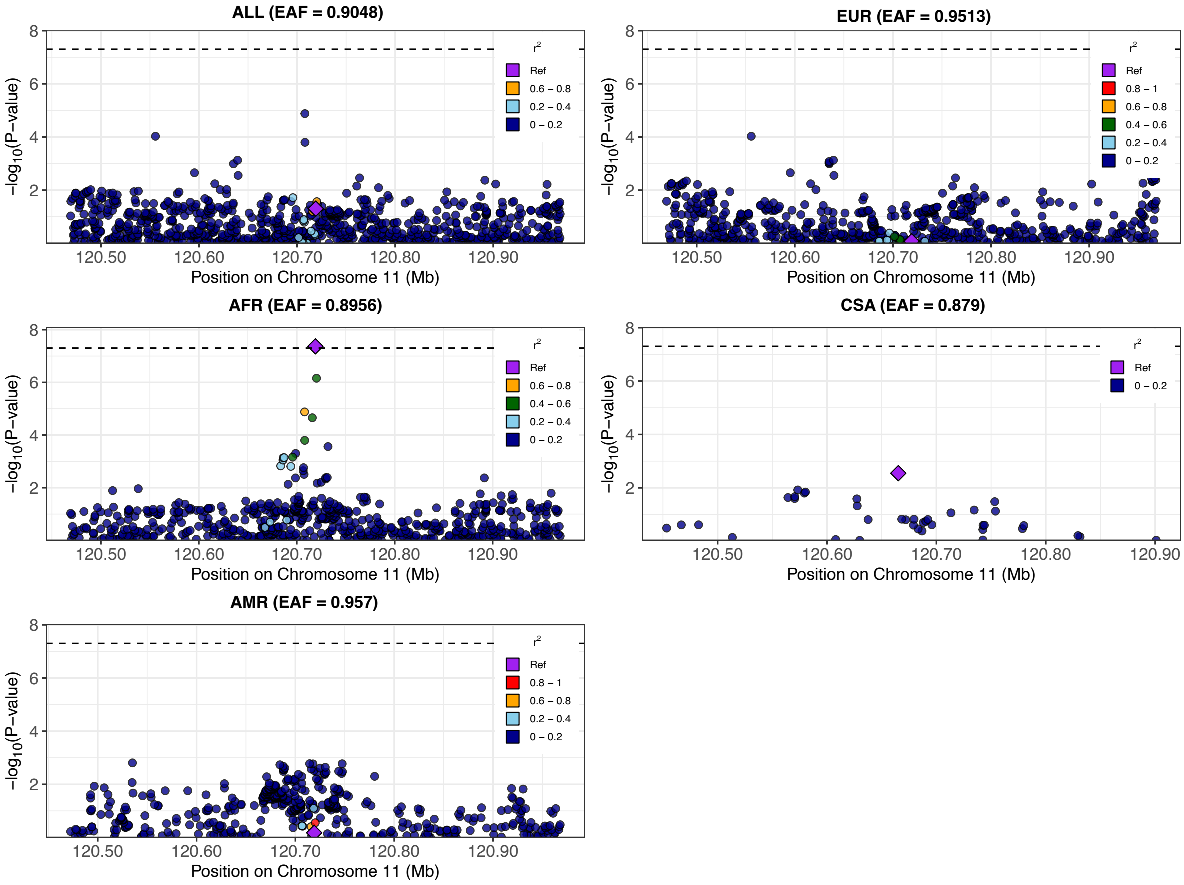

Supplementary Figure 2: Regional association plots for locus located nearest *GRIK4* where a genome-wide significant variant was identified in individuals of African ancestry.

medRxiv preprint doi: https://doi.org/10.64898/2026.05.12.26353033; this version posted May 15, 2026. The copyright holder for this preprint (which was not certified by peer review) is the author/funder, who has granted medRxiv a license to display the preprint in perpetuity.
It is made available under a CC-BY 4.0 International license .

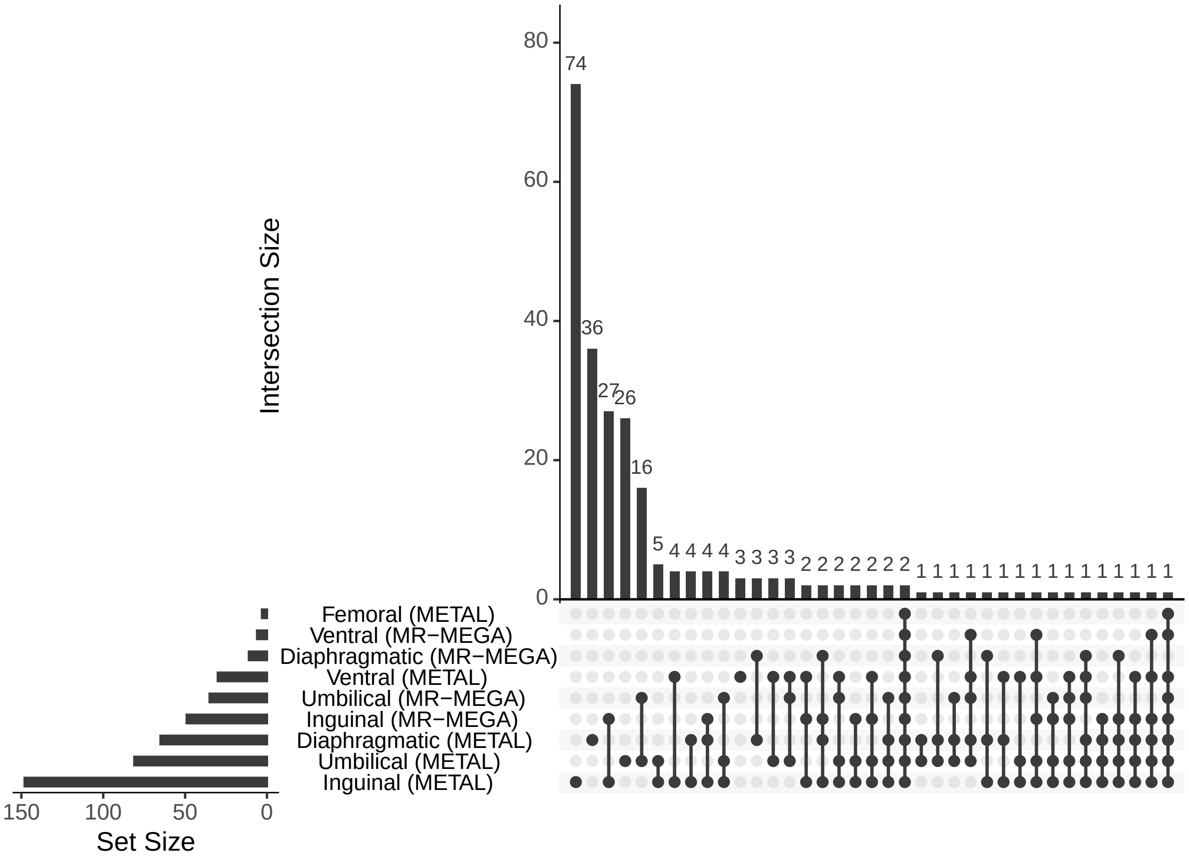

Supplementary Figure 3: Upset plot demonstrating the number of shared genome-wide significant loci between each hernia subtype and meta-regression method. Genome-wide significant loci were classified as shared between hernia subtypes if the 1 Mb region surrounding a lead variant overlapped with a 1 Mb region surrounding a different lead variant. All genome-wide significant loci identified by MR-MEGA were also present in the METAL results; however, MR-MEGA did not identify any novel loci beyond the METAL based regression.

medRxiv preprint doi: https://doi.org/10.64898/2026.05.12.26353033; this version posted May 15, 2026. The copyright holder for this preprint (which was not certified by peer review) is the author/funder, who has granted medRxiv a license to display the preprint in perpetuity.
It is made available under a CC-BY 4.0 International license .

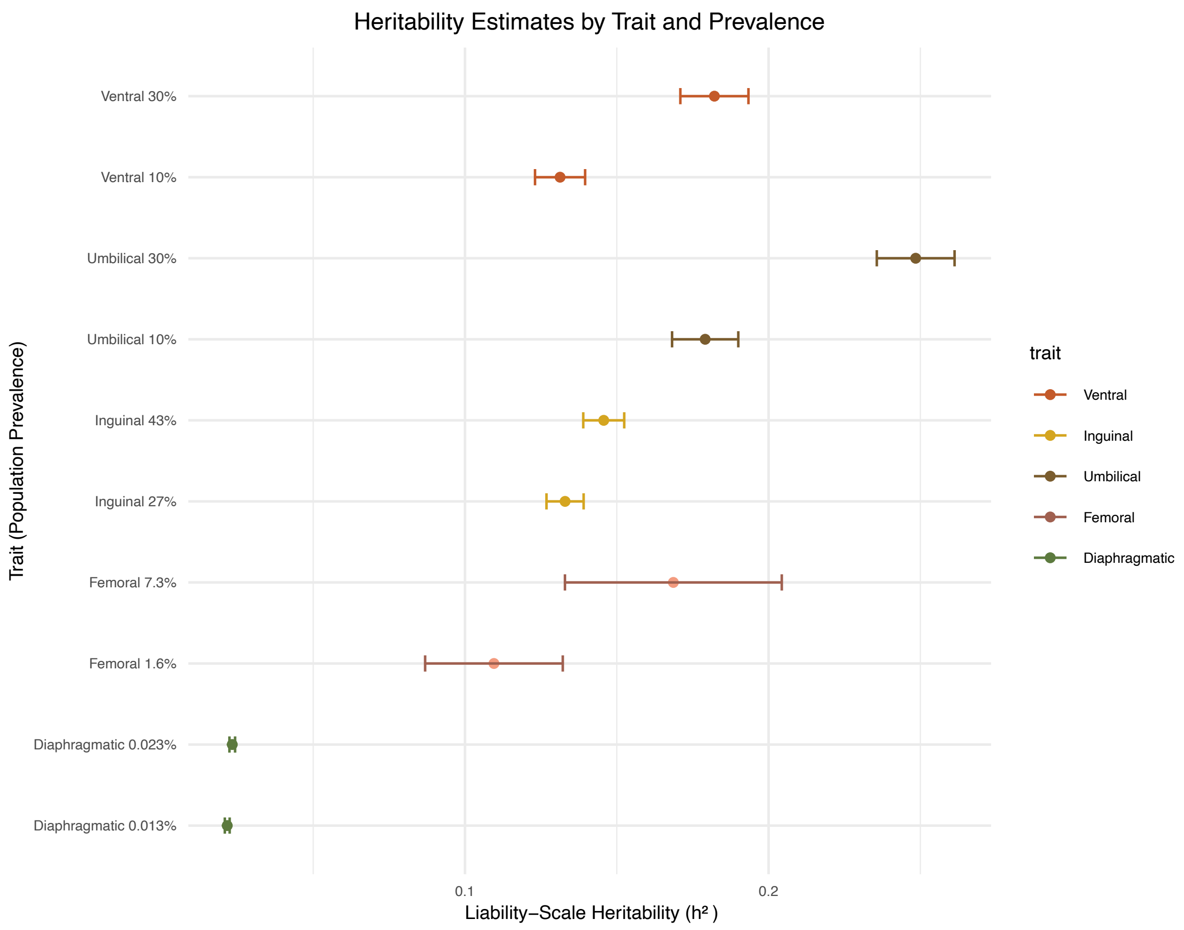

Supplementary Figure 4: Heritability estimates for each subtype of hernia. Linkage disequilibrium score regression was applied to multi-population meta-regression summary statistics to estimate observed scaled heritability. Observed scale estimates were converted to liability scale estimates using upper and lower bounds of population prevalences for each hernia subtype.

medRxiv preprint doi: https://doi.org/10.64898/2026.05.12.26353033; this version posted May 15, 2026. The copyright holder for this preprint (which was not certified by peer review) is the author/funder, who has granted medRxiv a license to display the preprint in perpetuity.
It is made available under a CC-BY 4.0 International license .

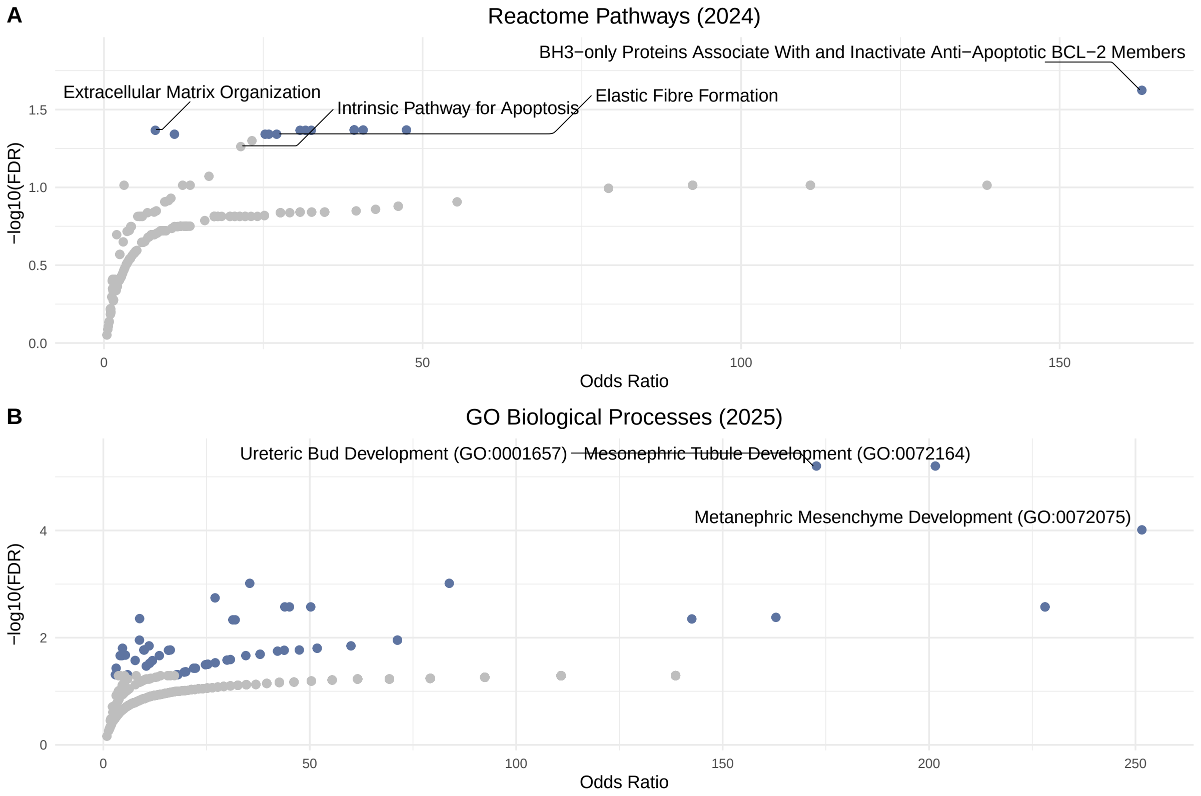

Supplementary Figure 5: Most enriched gene ontologies in Reactome Pathways (2024) and GO Biological Processes for protein-coding genes nearest to genome-wide significant loci demonstrating evidence for colocalization across hernia subtypes.

medRxiv preprint doi: https://doi.org/10.64898/2026.05.12.26353033; this version posted May 15, 2026. The copyright holder for this preprint (which was not certified by peer review) is the author/funder, who has granted medRxiv a license to display the preprint in perpetuity.
It is made available under a CC-BY 4.0 International license .

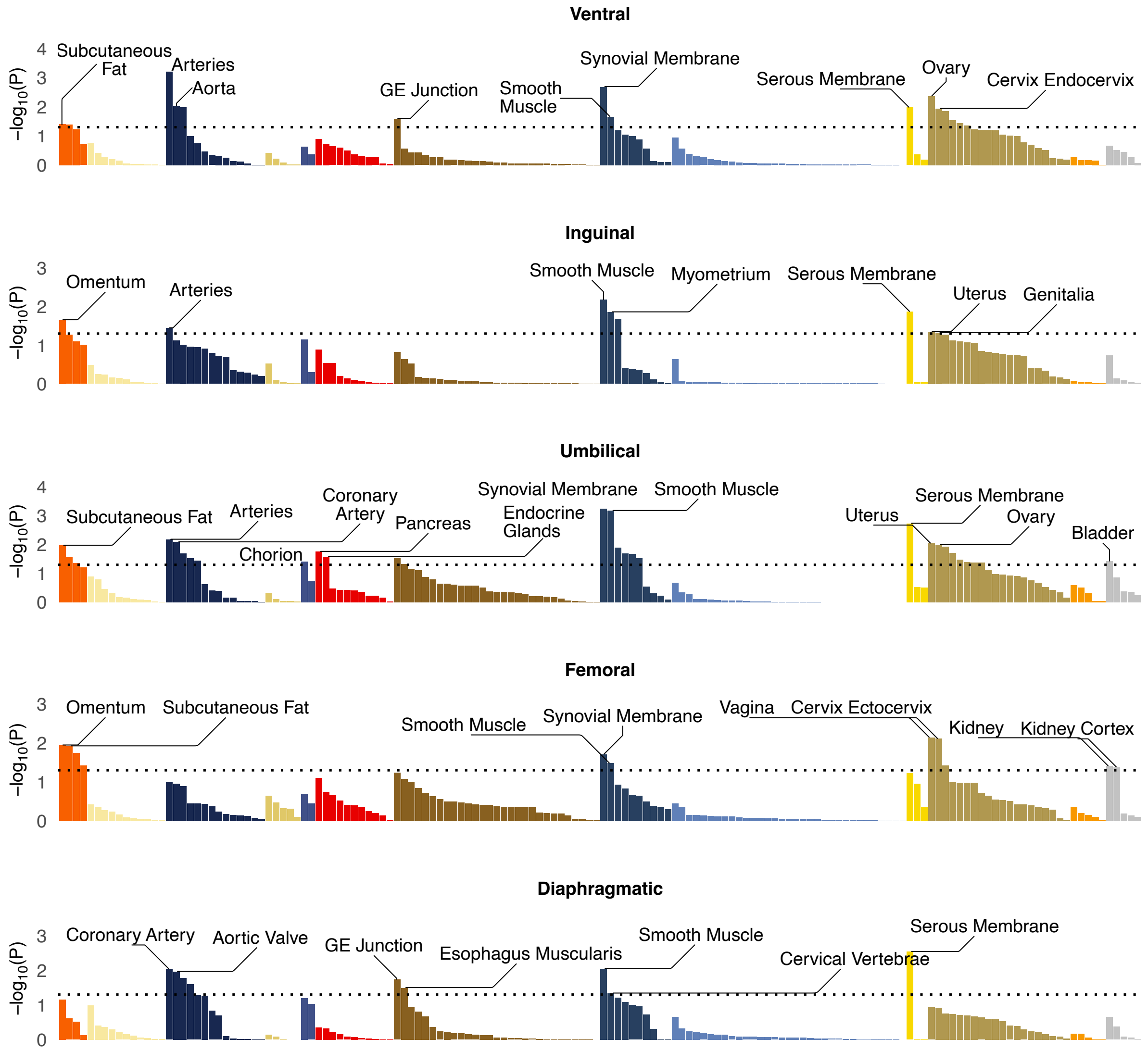

Supplementary Figure 6: Tissue enrichment analysis of hernia subtypes using LDSC-SEG. Enrichment for tissue-specific differentially expressed genes, showing strongest signals in adipose and vascular tissues. The dashed black line indicates the nominal significance threshold (P = 0.05). Tissue groups are color-coded by organ system.

medRxiv preprint doi: https://doi.org/10.64898/2026.05.12.26353033; this version posted May 15, 2026. The copyright holder for this preprint (which was not certified by peer review) is the author/funder, who has granted medRxiv a license to display the preprint in perpetuity.
It is made available under a CC-BY 4.0 International license .

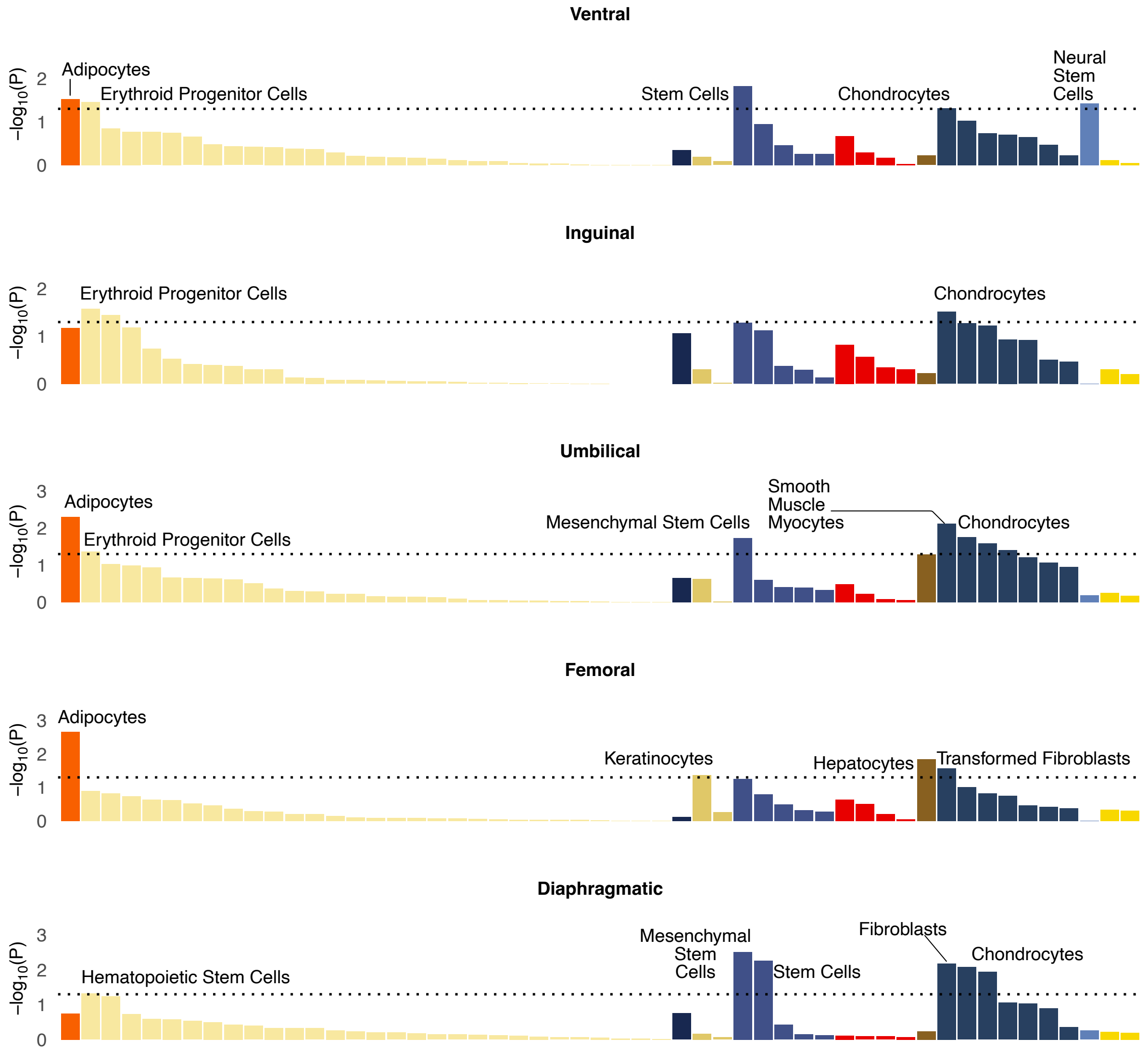

Supplementary Figure 7: Cell type enrichment analysis of hernia subtypes using LDSC-SEG. Enrichment for cell-specific differentially expressed genes, showing strongest signals in mesenchymal stem cells, chondrocytes, adipocytes, and fibroblasts. The dashed black line indicates the nominal significance threshold (P = 0.05). Tissue groups are color-coded by organ system.

medRxiv preprint doi: https://doi.org/10.64898/2026.05.12.26353033; this version posted May 15, 2026. The copyright holder for this preprint (which was not certified by peer review) is the author/funder, who has granted medRxiv a license to display the preprint in perpetuity.
It is made available under a CC-BY 4.0 International license .

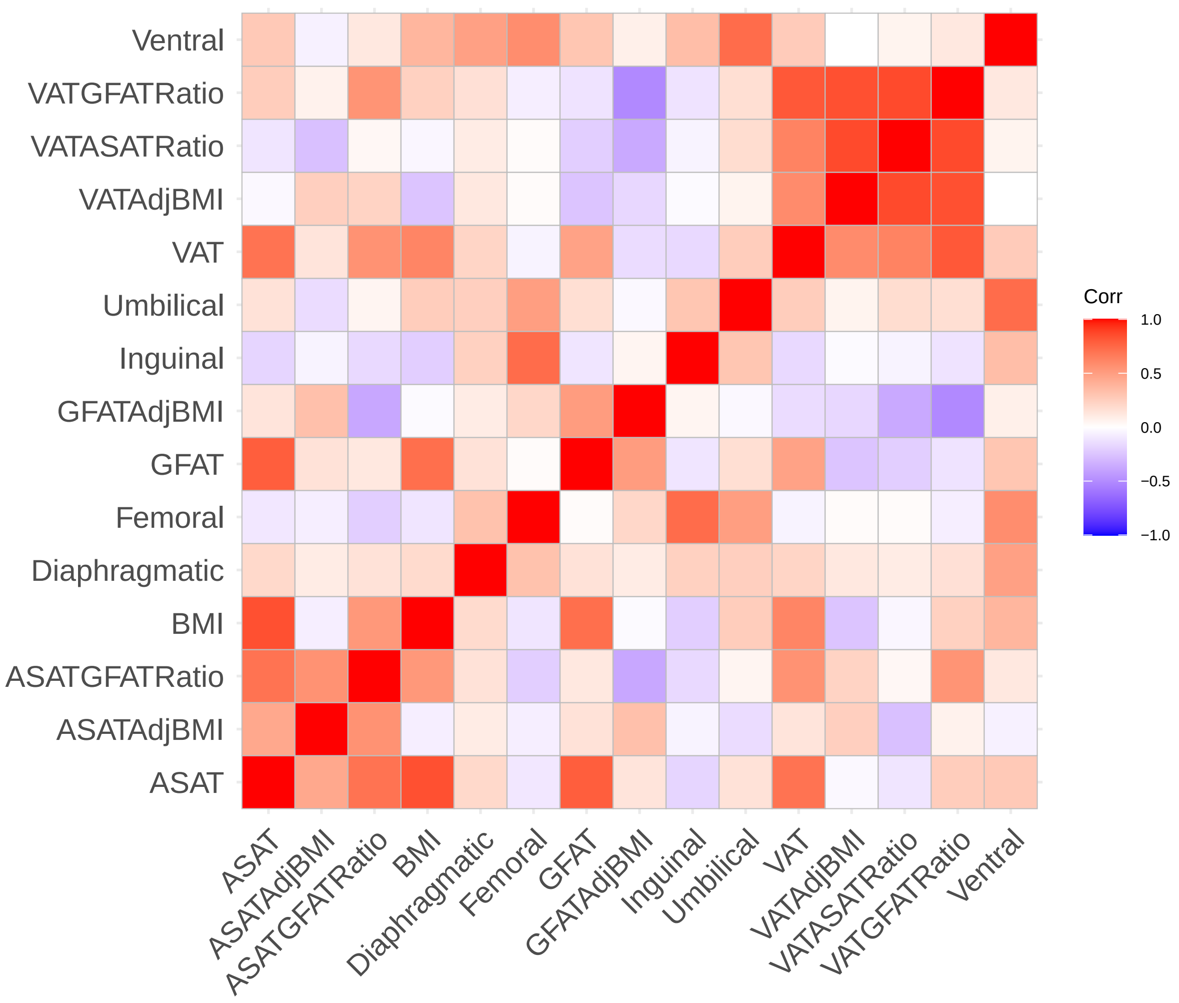

Supplementary Figure 8: Genomic correlation between hernia subtypes and anthropometric traits

medRxiv preprint doi: https://doi.org/10.64898/2026.05.12.26353033; this version posted May 15, 2026. The copyright holder for this preprint (which was not certified by peer review) is the author/funder, who has granted medRxiv a license to display the preprint in perpetuity.
It is made available under a CC-BY 4.0 International license .

Description of Supplementary Data

1. Number of participants with and without hernia subtypes, stratified by biobank and most similar 1000 Genomes reference population.
2. Lambda GC estimates and number of genome-wide significant variants for the meta-analyses, stratified by hernia subtype and population.
3. Lead variants identified in the multi-population meta analysis for each hernia subtype.
4. Credible sets containing less than ten variants for each hernia subtype.
5. Per-locus summary of variant-to-gene prioritization.
6. Gene set enrichment analysis of prioritized hernia effector genes using GO Biological Processes (2025).
7. Gene set enrichment analysis of prioritized hernia effector genes using the Reactome Pathways (2024).
8. Results of LAVA univariate analysis.
9. Results of LAVA bivariate analysis.
10. Results of Bayesian multi-trait colocalization analysis.
11. Statistical finemapping for causal variants at loci identified in colocalization analysis.
12. Gene set enrichment analysis of prioritized genes at loci identified in colocalization analysis
13. Results from exploratory factor analysis of hernia subtypes.
14. Summary details of Mendelian Randomization instruments.
15. Mendelian randomization instruments.
16. Mendelian randomization results.
17. Results of proteome-wide Mendelian randomization.
18. Results of Bayesian pQTL colocalization analysis.
19. Results of drug repurposing Mendelian randomization.
